# Application of Gold Nanoparticles as Radiosensitizer for Metastatic Prostate Cancer Cell Lines

**DOI:** 10.3390/ijms24044122

**Published:** 2023-02-18

**Authors:** Sílvia Soares, Isabel Faria, Fátima Aires, Armanda Monteiro, Gabriela Pinto, Maria Goreti Sales, Miguel A. Correa-Duarte, Susana G. Guerreiro, Rúben Fernandes

**Affiliations:** 1 ICBAS—School of Medicine and Biomedical Sciences, University of Porto, 4050-313 Porto, Portugal; 2 FP-I3ID, FP-BHS, Universidade Fernando Pessoa (UFP), 4249-004 Porto, Portugal; 3 Instituto de Investigação e Inovação em Saúde (i3S), 4200-135 Porto, Portugal; 4 Faculty of Chemistry, University of Vigo, 36310 Vigo, Spain; 5 CEB, Centre of Biological Engineering of Minho University, 4710-057 Braga, Portugal; 6 BioMark@ISEP/CEB—Center of Biological Engineering of Minho University, School of Engineering, Polytechnic Institute of Porto, 4249-015 Porto, Portugal; 7 School of Health, Polytechnic of Porto, 4200-072 Porto, Portugal; 8 Radiotherapy Service, São João Hospital Center, 4200-319 Porto, Portugal; 9 Biomark@UC/CEB—Centre of Biological Engineering of Minho University, Department of Chemical Engineering, Faculty of Sciences and Technology, Coimbra University, 3030-790 Coimbra, Portugal; 10 CINBIO, University of Vigo, 36310 Vigo, Spain; 11 Southern Galicia Institute of Health Research (IISGS), and Biomedical Research Networking Center for Mental Health (CIBERSAM), 36310 Madrid, Spain; 12 Institute of Molecular Pathology, Immunology of the University of Porto-IPATIMUP, 4200-465 Porto, Portugal; 13 Department of Biomedicine, Biochemistry Unit, Faculty of Medicine, University of Porto, 4200-319 Porto, Portugal; 14 Faculty of Health Sciences (FCS) & Hospital Escola Fernando Pessoa (HEFP), University Fernando Pessoa (UFP), 4249-004 Porto, Portugal

**Keywords:** gold nanoparticles, radiotherapy, radiosensitizing effect, prostate cancer, in vitro assay

## Abstract

More than 50% of all prostate cancer (PCa) patients are treated by radiotherapy (RT). Radioresistance and cancer recurrence are two consequences of the therapy and are related to dose heterogeneity and non-selectivity between normal and tumoral cells. Gold nanoparticles (AuNPs) could be used as potential radiosensitizers to overcome these therapeutic limitations of RT. This study assessed the biological interaction of different morphologies of AuNPs with ionizing radiation (IR) in PCa cells. To achieve that aim, three different amine-pegylated AuNPs were synthesized with distinct sizes and shapes (spherical, AuNP_sp_-PEG, star, AuNP_st_-PEG, and rods, AuNP_r_-PEG) and viability, injury and colony assays were used to analyze their biological effect on PCa cells (PC3, DU145, and LNCaP) when submitted to the accumulative fraction of RT. The combinatory effect of AuNPs with IR decreased cell viability and increased apoptosis compared to cells treated only with IR or untreated cells. Additionally, our results showed an increase in the sensitization enhancement ratio by cells treated with AuNPs and IR, and this effect is cell line dependent. Our findings support that the design of AuNPs modulated their cellular behavior and suggested that AuNPs could improve the RT efficacy in PCa cells.

## 1. Introduction

Radiotherapy (RT) is one of the most used therapeutic approaches to treat PCa, which is one of the most commonly diagnosed non-skin cancers affecting men, and in fourth place for cancer mortality in males [1]. Approximately 50% of all patients receive RT at some point during treatment [2]. RT is a therapeutic approach that uses ionizing radiation (IR) to induce cell damage and kill cancer cells. The main goal of this therapy is to deliver a precise dose of radiation to a tumor volume, thus promoting the irradiation of tumor cells with the minimum amount of damage possible to the surrounding healthy tissues [3]. 

One of the main challenges regarding the efficacy of RT is the presence of hypoxic tumor cells. These cells can contribute to the enhancement of radiation resistance due to the reduced production of reactive oxygen species (ROS) and, consequently, a decrease in oxidative stress and apoptosis [4]. Another clinical problem in PCa treatment is recurrence. Studies have shown that up to 50% of PCa patients suffer from radioresistance within five years after treatment because of the adaptation of the cell populations to RT [3,5,6]. Besides radioresistance, this well-establish cancer treatment is also limited in success by dose heterogeneity, local discomfort, and non-selectivity between normal and tumoral tissues, all of which results in unwanted side effects. Radiosensitizers and radioprotectors can be applied to avoid the increase in radiation doses and to overcome these adverse effects of IR [7].

RT can be associated with nanotechnology to overcome some of your limitations, which has the potential to improve the efficiency of therapies and reduce side effects on healthy tissues. Gold nanoparticles (AuNPs) can be used as radiosensitizers due to their properties [8,9,10]. High-atomic-element (Z) AuNPs have achieved widespread attention and have been considered a potential dose enhancing agent to RT [11,12]. Gold is a stable noble metal that is biocompatible and easily functionalized by many (bio)molecules such as polyethylene glycol (PEG) [13,14,15]. PEG is one of the most widely used biopolymers, and prevents immune recognition, stabilizes AuNPs, reduces the toxicity of nanoparticles, and improves the systemic circulation lifetime and the biocompatibility of nanocomplex [12,16]. 

AuNPs allow for strong attenuation of photons and, in addition to that, increase the maximal deposition of IR [17]. As a result, the absorbed energy leads to the emission of photoelectrons, Auger electrons, Compton electrons, and fluorescence photons that increase ionization of intracellular components or water molecules to generate ROS [18]. Therefore, functionalized AuNPs have aroused interest for clinical use such as therapy, image contrast agents, and diagnostic purposes [13,14,15]. The success of the interaction between AuNPs and IR could be related to some factors such as size, shape, surface chemistry, the concentration of AuNPs, and the targeted cell type [10,17,19,20,21]. Many studies have investigated the influence of the size of AuNPs on cells radiosensitization, but only a few report the interaction of different shapes of AuNPs with IR in vitro, and one article compared different shapes using Monte Carlo simulations [18,22,23,24]. 

To the best of our knowledge, this is the first study exploring the biological effect of AuNPs on radiosensitization on PCa cells. Therefore, to recognize the potential of AuNPs in RT, we synthesized several amine-pegylated AuNPs with different sizes and shapes (referred to as spherical gold nanoparticles, AuNP_sp_-PEG, gold nanostars, AuNP_st_-PEG, and gold nanorods, AuNP_r_-PEG) and analyzed their effect on PCa cells viability, migration, radiosensitivity, apoptosis/necrosis, and ROS production to understand whether those AuNPs can improve the RT efficacy by increasing the radiosensitization of the targeted cells.

## 2. Results

### 2.1. Characterization of AuNPs-PEG

**Figure 1:**
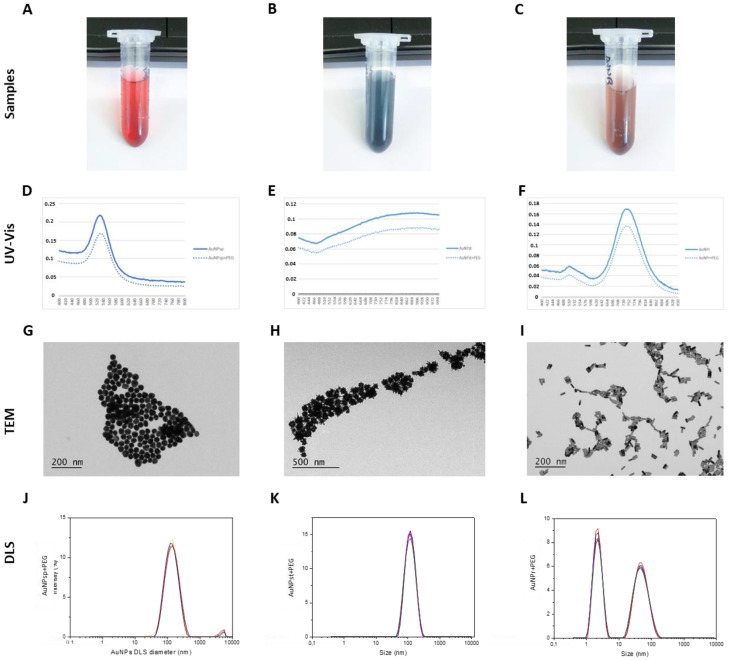
Characterization of AuNPs. (**A**–**C**) Visual appearance of samples; (**D**–**F**) UV-visible spectra of bare AuNP_sp_, AuNP_st_, AuNP_r_ (continuous line) and PEG-functionalized AuNPs, AuNP_sp_-PEG, AuNP_st_-PEG, and AuNP_r_-PEG (dashed line); (**G**–**I**) TEM imaging shows the morphology of the AuNPs; (**J**–**L**) The DLS peaks of AuNP_sp_-PEG, AuNP_st_-PEG, and AuNP_r_-PEG. AuNP_sp_-PEG, PEGylated spherical gold nanoparticles; AuNP_st_-PEG, PEGylated gold nanostars; AuNP_r_-PEG, PEGylated gold nanorods.

After linking AuNPs with PEG, the samples of AuNPs were characterized using a UV-visible spectrum, revealing characteristic surface plasmon resonance (SPR) absorption bands at approximately 533.2 nm, 909.8 nm, and 741.9 × 512.1 nm for AuNP_sp_-PEG, AuNP_st_-PEG, and AuNP_r_-PEG, respectively (Figure 1A–F). 

Additionally, the morphology of the nanoparticles was assessed by transmission electron microscopy—TEM (Figure 1G–I). AuNPs were evaluated using TEM images and dynamic light scattering (DLS) relative to hydrodynamic diameters. According to TEM analysis, the diameters for AuNP_sp_-PEG, AuNP_st_-PEG, and AuNP_r_-PEG were 48.20 ± 12.8 nm, 77.72 ± 16.05 nm and 36.10 ± 3.96 nm × 1.41 ± 1.48 nm (length × width), respectively. DLS analysis was used to determine the diameters of AuNP_sp_-PEG, AuNP_st_-PEG, and AuNP_r_-PEG, which were 146.73 ± 4.24 nm, 109.61 ± 1.27 nm, and 54.58 ± 0.34 nm × 8.47 ± 0.22 nm (length × width), respectively. The zeta potentials were −5.7 ± 7.6 mV, 33.1 ± 12.0 mV and 11.0 ± 18.9 mV, respectively, for AuNP_sp_-PEG, AuNP_st_-PEG, and AuNP_r_-PEG (Figure 1J–L and Table 1). 

Regarding the PDI of AuNPs, the AuNP_st_-PEG showed more monodispersity than AuNP_sp_-PEG and AuNP_r_-PEG, leading to more polydispersity (Table 1).

### 2.2. Contrast Effect of AuNPs-PEG Using CT

**Figure 2:**
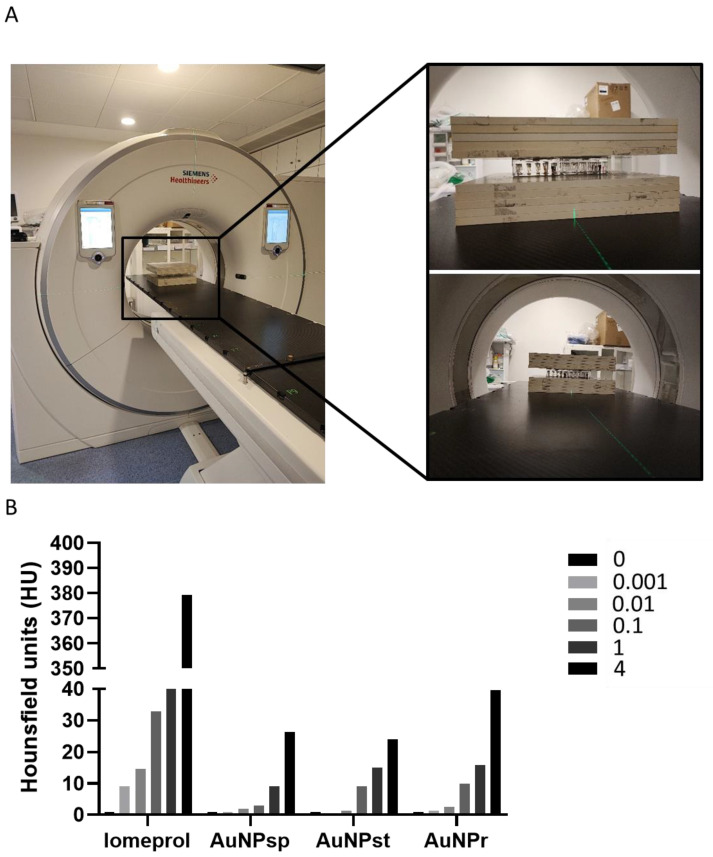
(**A**) Photos of the image acquisition set-up using clinical Light Speed VCT CT imaging system (GE Medical Systems, Milwaukee, WI, USA). (**B**) Representative histogram measurements of CT contrast attenuation rate values in Hounsfield units of different conformations of AuNPs under four different concentrations ([Au] = 0.001 to 4 mM) with PBS as control. Iomeprol was used as a reference of clinical practice. AuNP_sp_, spherical gold nanoparticles; AuNP_st_, gold nanostars; AuNP_r_, gold nanorods.

To study how the contrast effect of AuNPs were in CT, different concentrations of AuNPs (0.001 to 4 mM) were tested. It was found that AuNPs exhibited an attenuation factor tendency, especially for high concentrations (1 and 4 mM). Additionally, the attenuation factor tendency in CT contrast images was compared for different morphologies of AuNPs (Figure 2) and revealed that AuNP_r_ contributes to greater attenuation than AuNP_sp_ and AuNP_st_. 

For 4 mM, AuNPr showed greater X-ray attenuation than others, with CT values almost double those of the other AuNPs. 

### 2.3. Cellular Uptake of AuNPs-PEG

**Figure 3:**
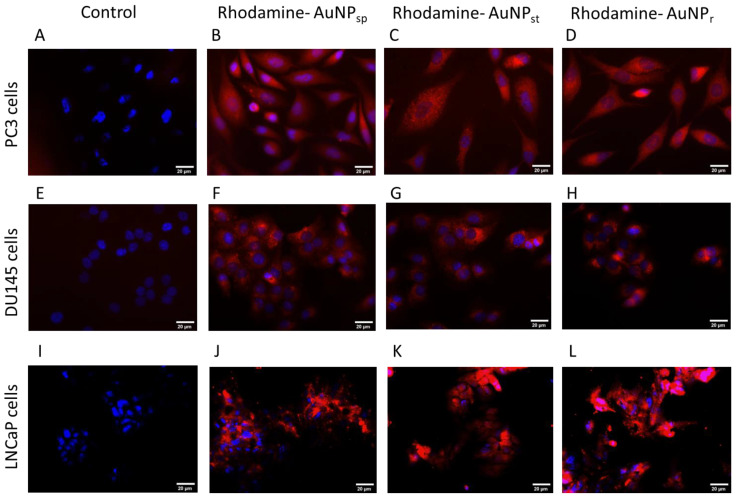
Fluorescence microscopy images of cellular uptake of AuNPs labeled with rhodamine B-labeled AuNPs in PC3 (**A**–**D**), DU145 (**E**–**H**) and LNCaP (**I**–**L**) cell lines. (**A**,**E**,**I**) were control groups (without treatment of AuNPs); (**B**,**F**,**J**) cells were treated with rhodamine-AuNP_sp_; (**C**,**G**,**K**) were treated with rhodamine-AuNP_st_; and (**D**,**H**,**L**) were treated with rhodamine-AuNP_r_. AuNPs-Rhodamine (red) cell nuclei were stained with DAPI (blue). AuNP_sp_-PEG, PEGylated spherical gold nanoparticles; AuNP_st_-PEG, PEGylated gold nanostars; AuNP_r_-PEG, PEGylated gold nanorods. Scale bars = 20 µm.

Cells were treated with different treatments of AuNPs during 24 h. After that, cellular uptake of AuNPs linked with rhodamine were observed by the red fluorescence signals inside of cellular cytoplasm, as shown in Figure 3.

AuNPs were found to be intracellular, indicating that AuNPs were internalized by endocytosis. It was observed that AuNPs were stored on cytoplasm for 24 h, in both cell lines analyzed. However, more studies should be performed to understand uptake via endocytosis. 

### 2.4. AuNPs-PEG Decreased Cellular Viability 

**Figure 4:**
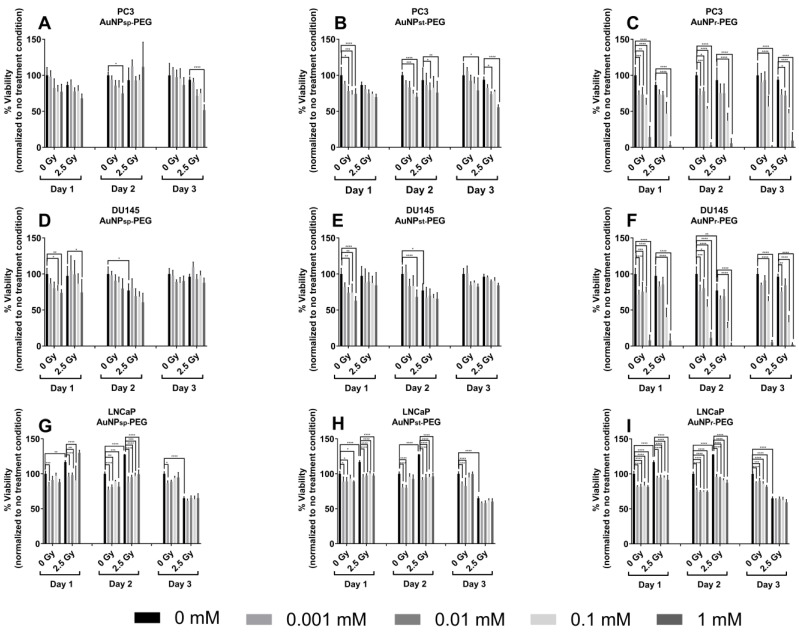
Effect of AuNPs and ionizing radiation (IR) on the viability of prostate cancer cell lines ((**A**–**C**), PC3; (**D**–**F**), DU145; (**G**–**I**) LNCaP). The cells were previously treated with different concentrations of AuNPs (0–1 mM), and then they were exposed to a cumulative dose of 7.5 Gy in three fractions of 6MV photon beam. An indication of cell viability was obtained by analyzing the overall metabolic activity of the cell population by PrestoBlue™ assay. Results were expressed as the mean ± SD, n = 6. AuNP_sp_-PEG, PEGylated spherical gold nanoparticles; AuNP_st_-PEG, PEGylated gold nanostars; AuNP_r_-PEG, PEGylated gold nanorods. The results were considered to be statistically significant when * *p* < 0.05, ** *p* < 0.01, *** *p* < 0.001 and **** *p* < 0.0001. The treatment groups were compared to the control group of the respective day, represented by black lines.

To evaluate the effect of AuNPs on cell viability, various cell lines were treated with different concentrations (0 to 1 mM) of AuNPs for 24 h to 72 h (Figure 4).

**Figure 5:**
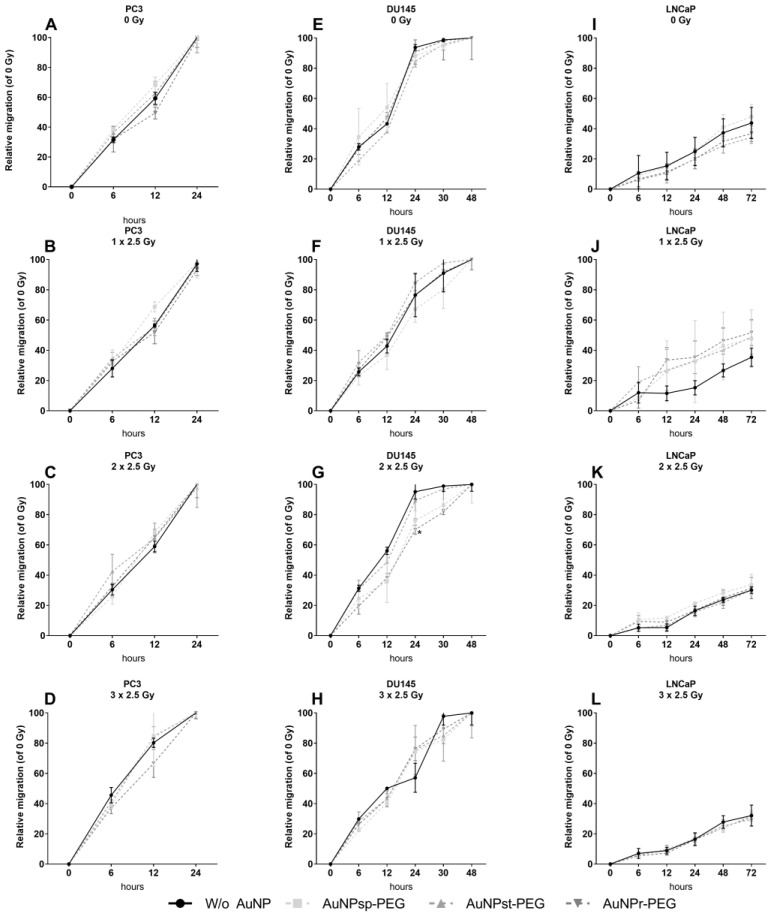
Effect of AuNPs and ionizing radiation (IR) on the wound healing of prostate cancer cell lines ((**A**–**D**) PC3; (**E**–**H**) DU145; (**I**–**L**) LNCaP). The cells were treated with different concentrations (0–1 mM) for 24 h before being exposed to a cumulative dose of 7.5 Gy in three fractions of 6 MV photon beam. Relative migration (%) was measured 0, 6, 12 and 24 h after scratching. The results are expressed as the mean ± SD of 3 replicates. AuNP_sp_-PEG, PEGylated spherical gold nanoparticles; AuNP_st_-PEG, PEGylated gold nanostars; AuNP_r_-PEG, PEGylated gold nanorods.

Cells were treated with different concentrations of AuNPs for 24 h. Then, the cells were exposed to a fraction of 2.5 Gy for three days, reaching a cumulative dose of 7.5 Gy of 6 MV photon beam. After 24 h of IR, the cell viability was measured, and the results are shown in Figure 5. We observed that most AuNPs were dose-dependent and did not increase the cytotoxic effects, except for 1 mM AuNP_r_-PEG. On day 1, AuNPs inhibited cellular viability with and without IR. On day 2, cells had more approximately 24 h to recover from the damages caused by the first IR. However, cells treated with AuNPs showed the capacity to repress the cell viability. On day 3, PC3 and DU145 cells demonstrated a tendency of reduced viability compared to the control group (0 Gy). Only DU145 cells treated with AuNP_sp_-PEG showed increased viability with 2.5 Gy. With respect to LNCaP cells, their viability decreased with AuNPs treatments compared to the control group in the first and second IR fractions. Comparing the three AuNP conformations used to treat the cells, AuNP_r_-PEG demonstrated viability reduction and dose dependence with repeated IR for three days. After this assay, the 0.1 mM concentration was selected to realize the next experiments. 

In an overview, the cells treated with the respective AuNPs until 0.1 mM maintained approximately 80% of cellular viability, supporting the negligible cytotoxicity towards the cells and suggesting their potential for RT therapeutic applications.

### 2.5. Injury Assay with AuNPs-PEG

This assay evaluated the effect of AuNPs on cell migration by comparing the initial and the final distance of the cell gap created on cell monolayers. Previously, the cells were treated with different conformations of AuNPs at 0.1 mM for 24 h. Then, the scratch of the cell culture was made. The gap closure was observed during 24 h, 48 h and 72 h for PC3, DU145, and LNCaP cells, respectively. After 24 h, the effect of the AuNPs on the gap size for each cell line was compared to the untreated control. As seen in Figure 5A–D, for the PC3 cell line, only AuNP_r_-PEG tended to delay the migration of cancer cells without receiving IR (~10%) and 24 h after the third fraction (3 × 2.5 Gy) by ~18%.

Regarding DU145 (Figure 5E–H), without IR, only AuNP_st_-PEG showed a tendency to decrease the migration by around 8%. When cells were submitted at two or three fractions of IR, AuNP_sp_-PEG, AuNP_st_-PEG, and AuNP_r_-PEG tended to exhibit reductions of 15%, 4.6%, 18% for 2 × 2.5 Gy and 9%, 4% and 6% for 3 × 2.5 Gy, respectively. In LNCaP cells (Figure 5I–L), AuNPs did not significantly influence migration, but without IR, AuNP_st_-PEG and AuNP_r_-PEG seemed to decrease migration by 4.7% and 4.4%, respectively. When cells were irradiated, the first fraction exhibited migration stimulation of ~13–21%, but stimulus decreased slightly with the second fraction, by around 2.7–5.6%, and it was AuNP_st_-PEG that corresponded to the highest reduction in the stimulus (not significant). After the third fraction of IR, no differences were observed when comparing the IR groups to the control groups.

### 2.6. Sensibilization Effect of AuNPs-PEG in PCa Cells

**Figure 6:**
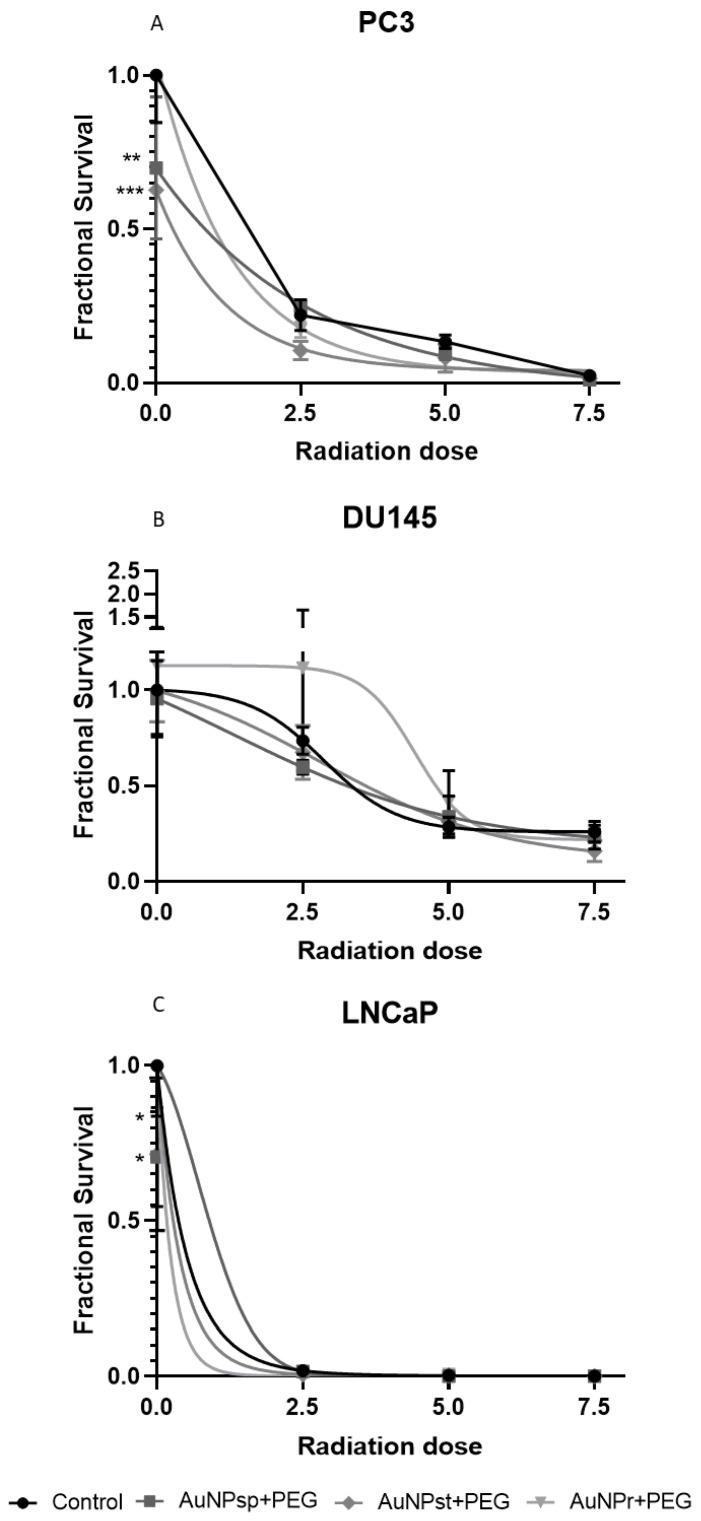
Survival curves of prostate cancer cells (PC3 (**A**), DU145 (**B**), and LNCaP (**C**)) plated immediately after respective RT treatments with fractions of 2.5 Gy until completion of a cumulative dose of 2.5, 5, and 7.5 Gy. The data presented are the mean ± SD of at least three independent experiments. The survival curves derived from clonogenic assay experiments are significantly different. AuNP_sp_-PEG, PEGylated spherical gold nanoparticles; AuNP_st_-PEG, PEGylated gold nanostars; AuNP_r_-PEG, PEGylated gold nanorods. Significance of different treatments compared to the control of the respective day shown as * *p* < 0.05, ** *p* < 0.01, *** *p* < 0.001.

After treatment with AuNPs for 24 h, radiosensitization was quantified using a clonogenicity assay. The results are shown in Figure 6.

In PC3 cells, treatment with either conformation of AuNPs tends to increase radiosensitization. However, in DU145, only cells treated with AuNP_sp_-PEG and AuNP_st_-PEG were shown to be slightly affected. For a cumulative 7.5 Gy dose, all AuNPs contributed to a slight decrease in the survival fraction. Treatment with AuNP_r_-PEG appeared to stimulate cell growth until cumulative 5 Gy. When LNCaP cells were treated with AuNP_sp_-PEG and AuNP_r_-PEG without IR, a reduction in the survival fractions was observed. After the first IR, LNCaP cells without AuNP treatment lost their capacity to grow into a colony. Regarding sensitivity enhancement ratio (SER) values (Table 2), for PC3 cells, all AuNP treatments caused an increase in cellular damage with IR, but AuNP_st_-PEG, AuNP_r_-PEG and AuNP_sp_-PEG produced a more significant increase in SER with doses of 2.5, 5, and 7.5 Gy (SER 2.09, 1.7 and 2.5, respectively), compared to the corresponding radiation group.

For DU145 cells, only AuNP_r_-PEG with 2.5 Gy exhibited a mild increase in sensibilization (SER 1.23) and all AuNPs showed an improved SER with 7.5 Gy (SER AuNP_st_-PEG—1.64; SER AuNP_r_-PEG—1.19 and SER AuNP_sp_-PEG—1.12) compared to the respective IR group. Moreover, LNCaP cells demonstrated a higher growth inhibition after 2.5 Gy with AuNP_st_ -PEG (SER 3) and a smooth increase with AuNP_sp_-PEG (SER 1.2) compared to the 2.5 Gy radiation group.

### 2.7. Apoptosis Assay with AuNPs-PEG

**Figure 7:**
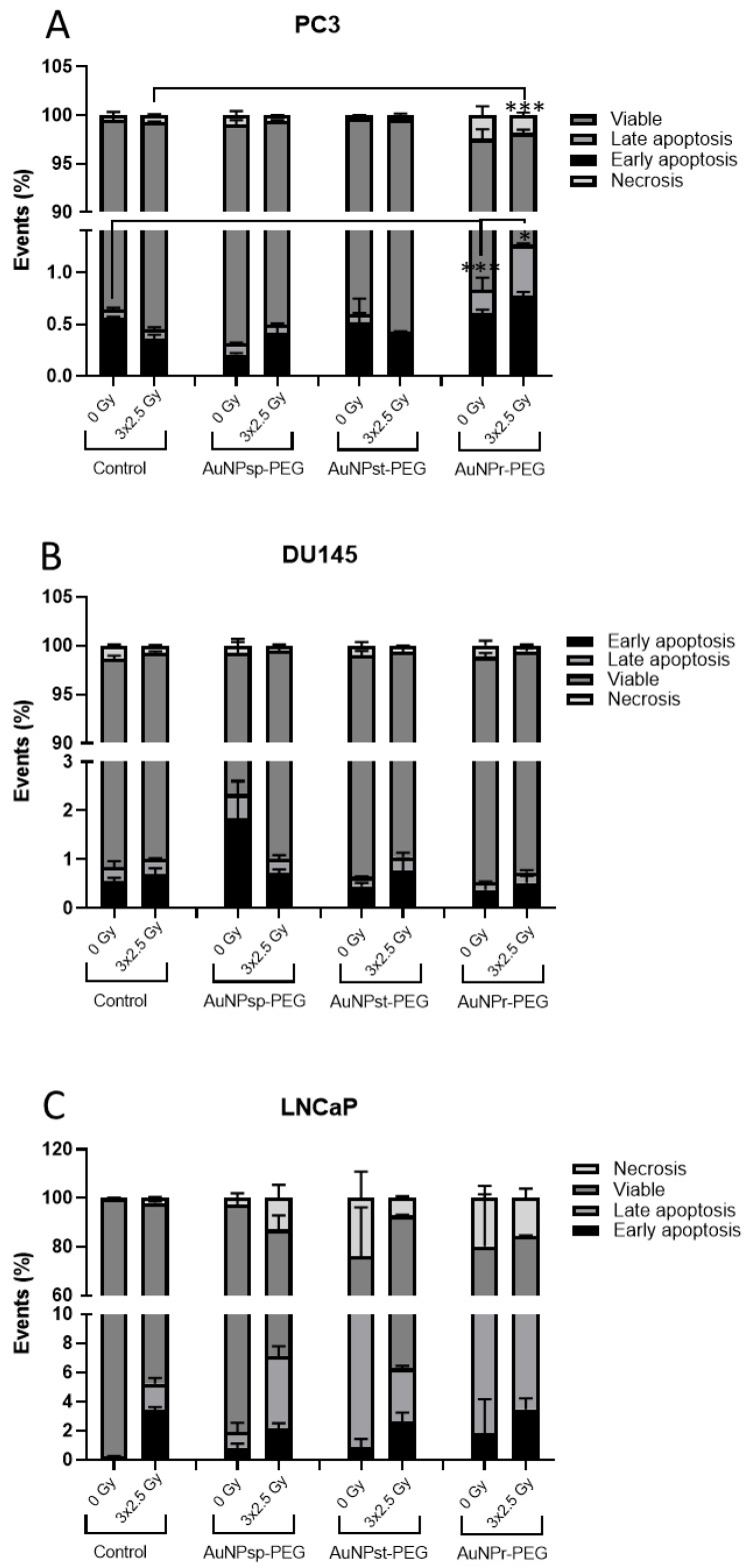
Cell apoptosis assay using annexin V–CF Blue/7-aminoactinomycin D (7-AAD). Graphs show the quantification of the percentage of apoptotic cells in PC3, DU145, and LNCaP cells. The results were obtained using flow cytometry assay after Annexin V/7-AAD staining and average percentage of apoptotic cells in (**A**) PC3, (**B**) DU145, and (**C**) LNCaP cells treated with different conformations of AuNPs—spherical (AuNP_sp_-PEG), star (AuNP_st_-PEG), and rod (AuNP_r_-PEG) and irradiated with 3 fraction of 2.5 Gy of RT. The obtained results were compared to the respective control groups with and without IR and are presented as mean ± SD. AuNP_sp_-PEG, PEGylated spherical gold nanoparticles; AuNP_st_-PEG, PEGylated gold nanostars; AuNP_r_-PEG, PEGylated gold nanorods. The significance of the different treatments compared to the control of the respective day are shown as * *p* < 0.05, *** *p* < 0.001.

The influence of AuNPs on cellular death with and without IR was evaluated using Annexin V–CF Blue/7-aminoactinomycin D (7-AAD)—Figure 7.

In PC3 cells, only AuNP_r_-PEG significatively reduced the cellular viability and increased the death cell percentage with IR (3.2%) and without IR (3.1%) compared to the respective control groups. When cells were treated with AuNPs, necrosis events were also observed. For AuNP_sp_-PEG and AuNP_st_-PEG, the cellular death values were similar to the control groups. Regarding DU145, the results showed a tendency for AuNP_st_- PEG to increase cellular death through apoptosis without IR. However, when cells were submitted to IR, the cellular death results were similar to the control group. Concerning the LNCaP cells, all AuNPs increased cellular death, mainly by necrosis without IR treatment, while with IR, the increase in cell death seems to be mainly induced by apoptosis. 

### 2.8. ROS Assay with AuNPs-PEG

**Figure 8:**
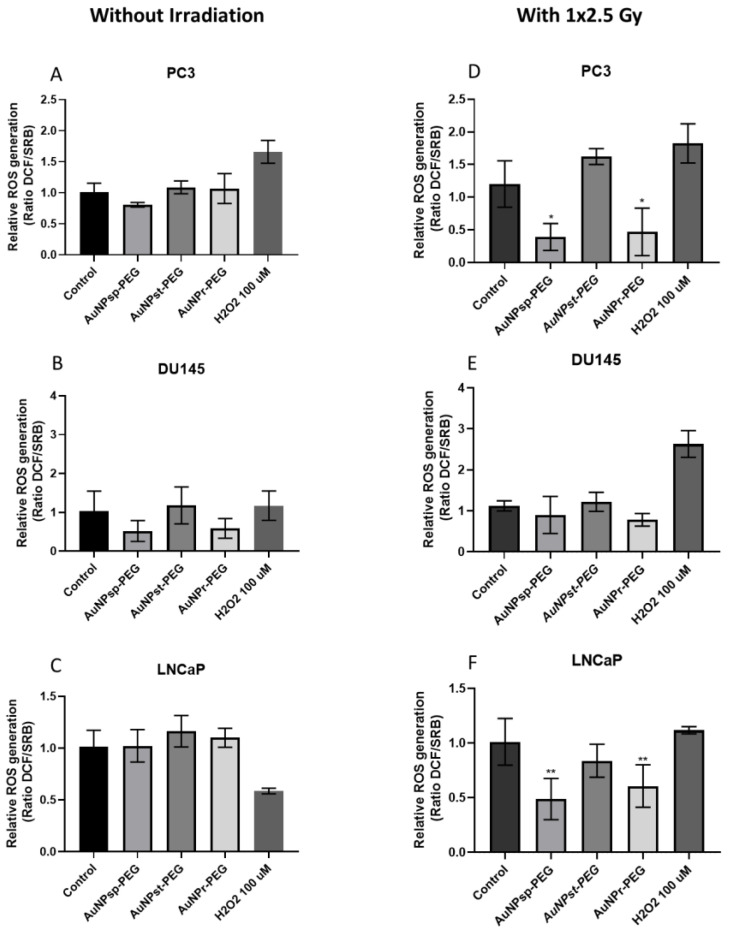
Relative mitochondrial ROS levels in (**A**,**D**) PC3, (**B**,**E**) DU145, and (**C**,**F**) LNCaP cells before (**A**–**C**) and after 2.5 Gy of RT (**D**–**F**) without AuNPs and with AuNPs. The measurements were taken 24 h after treatment. H_2_O_2_ was used as positive control. Data are presented as mean values ± SD of five replicate samples. AuNP_sp_-PEG, PEGylated spherical gold nanoparticles; AuNP_st_-PEG, PEGylated gold nanostars; AuNP_r_-PEG, PEGylated gold nanorods. Significance of different treatments compared to control of the respective day are shown as * *p* < 0.05, ** *p* < 0.01.

Overgeneration of ROS may cause DNA damage during mitochondrial respiration. ROS levels were detected by H2DCFDA to determine mitochondrial damage during exposure to AuNPs and IR. Figure 8 shows that AuNPs tended to increase ROS production in PC3 and LNCaP cells; however, the results were not statistically significant. 

Otherwise, AuNP_sp_-PEG and AuNP_r_-PEG showed a tendency to decrease ROS production in DU145 cells. However, when PC3 cells were irradiated with 2.5 Gy, AuNP_sp_-PEG and AuNP_r_-PEG contribute to reduce ROS production significatively and AuNP_st_-PEG demonstrated a predisposition to increase the ROS production but was not significant. Additionally, AuNP_sp_-PEG and AuNP_r_-PEG exhibited a tendency to decrease ROS production in the DU145 cell line. Regarding LNCaP cells, AuNP_sp_-PEG, AuNP_st_-PEG, and AuNP_r_-PEG revealed a decreased of ROS production, but only AuNP_sp_-PEG and AuNP_r_-PEG had statistical significance.

## 3. Discussion 

In the literature, only a few studies have explored the biological interaction between different conformations (shapes/sizes) of AuNPs and IR [17,25]. The most widely used PCa epithelial cell lines in RT studies are PC3, DU145 and LNCaP, being derived from PCa bone, brain, and lymph node metastases, respectively [26]. Regarding radiosensitivity, LNCaP appears to be the most radiosensitive, followed by DU145 and PC3 cell lines [27,28]]. Therefore, analyzing different types of cells could help to obtain results more representative of a wide range of PCa, because tumors are known to be heterogeneous, and tumor cells could have distinct features, such as aggressiveness and hormonal dependence. To the best of our knowledge, this is the first study comparing the effect of different conformations of PEGylated AuNPs on PC3, DU145, and LNCaP cells with cumulative doses of IR.

Different sizes and shapes of AuNPs have been explored for their potential to enhance RT, but there is still no consensus.

The most commonly studied AuNPs in this context are AuNP_sp,_ and Dou et al. suggested that AuNPs with a size of 3–50 nm are in the optimal size range for CT imaging and RT [22]. It has been reported that AuNPs of ~13 nm can be used in clinical X-ray theranostic applications and have a better effect on tumor growth [29,30]. The radiosensitization depends on, in addition to size and shape, the number of internalized AuNPs [31]. Chithrani et al. demonstrated that 50 nm AuNPs have the best cellular uptake in terms of both weight and number [32]. Additionally, other shapes such as AuNP_r_, AuNP_st_, triangles, and cubes have also been studied due to their unique optical and physical properties [10,18,23]. Therefore, the optimal size and shape of AuNPs for RT potentiation are still under investigation and may depend on the specific application and mode of action. 

Our results from CT imaging demonstrated that AuNPs’ attenuation factor was directly proportional to AuNP concentration. Studies have proposed that AuNPs could be a good candidate as contrast for CT imaging [33,34]. Our results are in accordance with literature results studying a concentration of contrast from 0 to 1 mM [22,34,35]. Previously, studies have reported conflicting results regarding the influence of the size of AuNPs as a contrast agent, but no information was found regarding the shape of AuNPs [22,33,36].

On the basis of TEM images, the intracellular biodistribution of AuNPs tested in cell cytoplasm was demonstrated. Size, shape, surface chemistry, and surface modifications of AuNPs have an important role in biodistribution [10,17,37,38]. A recent study indicates that mitochondrial damage also presents a risk to long-term cancer cell growth [39]. 

Herein, the effect on cellular viability was evaluated after different treatments of AuNPs-PEG, in which the size, shape, and concentration of AuNPs were variable. Our results indicated lower cytotoxicity levels of AuNP treatments in the three cell lines, as shown in Figure 4, and in agreement with other studies [13,40,41]. The effect of AuNP conformation on cellular viability differs from PC3, DU145 and LNCaP cells, which could have different endocytosis capacity of AuNPs per unit volume. LNCaP cells have lower cellular volume and are less radioresistant than PC3 and D145 cell lines [20]. Therefore, the efficacy of cell endocytosis/uptake of AuNPs could influence the results. Of the concentrations tested, it was decided to use 0.1 mM for the following experiments: migration, survival curve, apoptosis, clonogenic and ROS assays [30,42].

Our results showed a tendency to reduce migration, which is in accordance with the literature [43,44,45,46]. Cell migration could be affected by various AuNP characteristics, such as size, shape, and surface chemistry. AuNPs could interfere with cellular signaling pathways that affect cell adhesion, and actin cytoskeleton dynamics, leading to senescence or cell death [47,48]. Further research is needed to fully understand the impact of AuNPs on cell migration and the underlying mechanisms involved. 

The clonogenic assay is widely used to investigate cell damage induced by radiation [49,50]. Our results showed that AuNPs improved the radiosensitivity of PCa cells (Figure 6 and Table 2).

Some articles have shown that AuNP_sp_ inhibit colony forming ability in pancreatic cancer cells, but others have identified no long-term effects on colony formation in PC3, DU145 and MCF-7 cell lines [51,52]. Concerning IR, AuNP_sp_ (~12 nm) decreased the number of colonies formed when irradiated with 2 Gy. At the same time, non-irradiated cells treated with different concentrations of AuNP_sp_ had a similar result in the control (untreated cells) [53]. Similarly, Zhu et al. combined X-ray (1 to 8 Gy) with simple AuNP_sp_ (~20 nm) or modified them with galactose-pegylated AuNP_sp_ (GAL-(SH-PEG-NH_2_)-AuNP_sp_ (~34 nm) and both structures inhibited the colony formation, but the GAL-PEG-AuNP_sp_ showed better results, indicating that it could enhance the radiation sensitivity of HepG2 cells to X-ray [54]. Moreover, the interference of AuNPs on cell signaling pathways could lead to cellular damage and consequently contribute to reduced colony formation. However, more studies should be performed to identify how AuNPs affect colony formation [47]. 

It was verified that all treatments with different conformations of AuNPs at 0.1 mM induced more cell death through apoptosis or necrosis. AuNP_r_-PEG seems to increase apoptosis in all cell lines analyzed. In addition, these results are in agreement with the viability results obtained for these cell treatments. AuNPs may activate apoptotic pathways through a variety of mechanisms, including the release of cytotoxic molecules and the activation of specific signaling pathways [55,56,57]. In general, the effect of AuNPs on cell death is a complex mechanism, and it is not fully understood yet [57]. 

Regarding ROS production, cells without IR showed no significant differences between AuNPs-PEG tested. However, our results revealed that most AuNPs, especially AuNP_sp_-PEG and AuNP_r_-PEG, decreased the ROS production in PC3 and LNCaP cells.

In the literature, some studies support the notion that AuNPs could have antioxidant properties decreasing ROS production, while other studies have said that AuNPs could increase ROS production in the cell. One way that AuNPs may decrease ROS production is by inducing cells to employ many enzymatic and non-enzymatic antioxidants to neutralize the effect of ROS and bring back cell homeostasis [52]. Another hypothesis is that AuNPs may also scavenge ROS directly, effectively balancing them and reducing their levels in cells [53,54,58]. Overall, the available evidence suggests that AuNPs may be a promising strategy for reducing oxidative stress and mitigating the damaging effects of ROS depending on the conformation of AuNP used. On the other hand, human epidermal keratinocyte cells (HaCaT) treated with AuNP_r_-PEG (16.7 nm × 43.8 nm) produced significant ROS production when compared to mercaptopropane sulfonate (MPS)-AuNP_sp_ (20 nm), leading to an upregulation of apoptosis-related genes (TNFSF10, ANXA5, CASP1, and EGR1) and proteins (caspase 1) [59]. Another study verified that Calu-3 epithelial cells treated with hexagonal-AuNPs generated more ROS and pro-apoptotic markers (Fas, caspase 3, and caspase 9) when cells were treated with triangular or spherical AuNPs [60]. Further research is needed to fully understand how AuNPs can induce cell death and optimize their use in various applications. 

Among the published articles, it is difficult to make a meaningful comparison between studies because there are many variables related to nanoparticles and radiation parameters, cell types and experimental methodologies. Our study showed promising outcomes in using AuNPs as radiosensitizers, which is consistent with previous reports [18,20,30,55,61,62,63,64]. Additionally, our irradiation methodology differs from other published articles, because our irradiation scheme was divided into three cumulative fractions of 2.5 Gy in order to be able to bring our study to actual clinical RT treatment and understand the authentic behavior of AuNPs during the standard treatment of PCa. Typically, in vitro studies perform single irradiation with different doses, with most studies not reporting on cumulative doses [18,19,30,32].

## 4. Materials and Methods 

### 4.1. Chemicals

Trisodium citrate dehydrate (C_6_H_5_O_7_Na_3_ ·2H_2_O or NaCt), silver nitrate (AgNO_3_), tetrachloroauric acid tetrahydrate (HAuCl_4_^.^4H_2_O; 99.99% trace metals basis), Thiol-polyethylene glycol-amine (SH-PEG-NH_2_, molecular weight 2 kDa), fetal bovine serum (FBS), phosphate buffered saline (PBS) and trypsin were purchased from Sigma Aldrich^®^ LLC, St. Louis, MO, USA; Roswell Park Memorial Institute (RPMI) media and Minimum Essential Medium (MEM) were purchased from Biowest^®^; and PrestoBlue™ cell viability reagent (PB) were obtained from Invitrogen Co. (Scotland, UK); Annexin V-CF Blue 7-AAD apoptosis staining/detection kit (ab214663) from Abcam; DCFDA/H2DCFDA—Cellular ROS Assay Kit (D399) from Invitrogen; and QIAzol from Qiagen.

### 4.2. Synthesis of AuNPsp-PEG, AuNPst-PEG and AuNPr-PEG and Characterization

AuNP_sp_ were prepared using the method of Turkevich and his co-workers, using a HAuCl_4_.4H_2_O solution reduced and stabilized by NaCt [65]. AuNP_st_ were obtained according to the protocol reported by Tian et al. using a principle of growing a seed solution with AgNO_3_ and L-ascorbic acid [66]. Similarly, AuNP_r_ were produced using a seed growth solution based on the work of Scarabelli et al. After synthesis, PEGylation was achieved by adding SH-PEG-NH_2_ to the AuNPs solution [67]. After 24 h stirring, the solution was washed twice at 7500 rpm for 30 min.

After synthesis, samples were analyzed using an Evolution 200 Series spectrophotometer UV-VIS spectrophotometer (ThermoFisher Scientific Inc., USA). The absorption values were used to determine the concentration of species in the solution. Additionally, samples were examined by transmission electron microscopy (TEM, JEOL JEM 1400 TEM at 120 kV, Tokyo, Japan) and scanning electron microscopy (SEM) using FEI Quanta 400FEG ESEM/EDAX PEGASUS X4M equipment to validate the synthesis and morphology of AuNPs. Furthermore, the nanoparticles’ size distribution and zeta potential were analyzed by dynamic light scattering (DLS) and zetasizer (Nano ZS, Malvern Instruments Ltd., Malvern, UK), keeping the samples at 25 °C. 

### 4.3. In Vitro Attenuation Measurement in CT

Pegylated AuNPs were diluted in PBS at different concentrations of Au or iodine (reference group) from 0 to 4 mM. Iomeprol is a nonionic, monomeric iodinated contrast medium used in clinical practice. Air and PBS were used as control [68]. Samples were placed in 0.5 mL Eppendorf tubes, and CT images were obtained using a clinical Light Speed VCT CT imaging system (GE Medical Systems, Milwaukee, WI, USA). CT scanning parameters were the same as clinical practice for the abdominal area: slice thickness, 1.0 mm; pitch, 0.8; tube voltage, 120 kV; tube current, 101 mA; field of view, 500 × 500; and gantry rotation time, 1 s. Subsequently, attenuation measurements were evaluated by loading the digital CT images in a standard display program, allowing us to quantify the Hounsfield units (HU) related to CT contrast values. 

### 4.4. Cell Culture 

The PC3, DU145, and LNCaP cell lines used in this study were kindly donated by Cancer Biology and Epigenetics Group—Research Center, Portuguese Oncology Institute of Porto, Portugal. PC3 and LNCaP cells were cultured and maintained in RPMI-1650 media (Biowest^®^) and DU145 cells are maintained in MEM media (Biowest^®^), supplemented with 10% of fetal bovine serum (FBS, Sigma) and 1% penicillin/streptomycin (Sigma). Both cell lines were cultured and grown to ~80% confluence and were then sub-cultured and maintained at 37 °C with 5% CO2 in a humidified environment. All treatments were performed in serum-free conditions.

#### 4.4.1. Cellular Uptake Experiments

Rhodamine (20 mM) was conjugated with AuNPs and incubated for 2 h. Subsequently, AuNPs were centrifugated and washed three times to remove the excess. Rhodamine B isothiocyanate is a red fluorescent dye (Ex 560 nm/ Em 580 nm) that can be applied as a marker for a cell′s endocytic activity. Cells were treated with 0.1 mM Rhodamine-labeled AuNPs for 24 h. Then, the cells were observed via fluorescence microscopy (Carl Zeiss, Germany) with Zeiss Axio Imager Z1 software. The nuclei of the cells were stained with blue fluorescent 4′,6′-diamino-2-fenil-indol (DAPI, blue) and cells without rhodamine-tagged particles were used as the negative control for the studies. 

#### 4.4.2. Cell Viability 

The viability of cells treated with AuNPs was assessed by PrestoBlue™ assay. This allowed an indirect estimation of cell viability, due to resazurin being converted to resofurin by mitochondrial activity [69].

Cells were seeded (1 × 10^5^ cells/mL) in 96-well plates. After 24 h, cells were exposed to several concentrations of AuNPs ranging from 0 to 1 mM for another 24 h. Cells were exposed to 6 MV photon beam with a dose per fraction of 2.5 Gy, and the process was repeated for three days until cells had received a total cumulative dose of 7.5 Gy, except for the control group. After the treatment, 10% (*v/v*) PrestoBlue™ reagent was added per well and incubated for 1 h at 37 °C in a humidified incubator. The absorbance was measured using a microplate reader (Spectra Max Gemini XS) at 550 nm.

#### 4.4.3. Cell Migration

Cell migration may be assessed in vitro by wound injury assay. After reaching confluence, cells were scraped from the culture dish using a pipette tip. Then, cells were incubated for 24 h with different treatments, and the damage recovery was visualized and photographed under an inverted microscope (Nikon) at 200× magnification. Wound closure was measured at 0 h, 6 h, 12, 24 h and 48 h using ImageJ software (U.S. National Institutes of Health) and calculated using Equation (1):

(1) $$Wound\;area\left(\%\right)=\frac{Area\;at\;0h-Area\;at\;\left(x\right)h\;(treated\;cells)}{Area\;at\;0h\;(control)}\times 100$$

#### 4.4.4. Colony Assay and Sensitization Enhancement Ratio (SER)

Clonogenic assay (colony formation) is an in vitro cell survival assay based on the ability of a single cell to form a colony which consists of at least 50 cells. Clonogenic assay was performed to compare the effect of AuNPs and RT on cell death. Thus, cells were seeded, treated for 24 h, and irradiated the following day with a single dose of 2.5 Gy for three consecutive days using a linear accelerator until it reached a cumulative dose of 7.5 Gy. Immediately after IR, 250–1500 cells were seeded in each well and incubated at 37 °C with 5% CO_2_ for 7 days. After colony formation, cells were washed, fixed with 4% (*v/v*) paraformaldehyde, and stained with 5% crystal violet (*v/v*). Colonies with at least 50 cells were counted manually and surviving fractions (SF) at each dose were calculated using Equation (2):

(2) $$Survival\;fraction\;\left(SF\right)=\frac{\frac{No.colonies\;formed}{No.of\;cells\;seeded}\left(treated\right)}{\frac{No.colonies\;formed}{No.of\;cells\;seeded}\left(control\right)}$$

The experimental data of cell survival were fitted using the linear quadratic (LQ) model using Prism 8.0 (GraphPad Software, CA, USA). The parameters from the LQ formula given in Equation (3) were analyzed, where D is the physical dose expressed in Gy, and α and β are the model constants.

(3) $$SF=e^{-(\alpha D{-\beta D}^{2})}$$

Additionally, the radiosensitization effect of AuNPs on RT was evaluated by calculation of sensitizer enhancement ratio (SER, Equation (4)). The SER was calculated as the quotient of the survival fraction without AuNPs treatment and of that in the presence of AuNPs. The greater the SER, the greater the destruction of PCa cells in the presence of AuNPs at the same radiation dose, leading to a reduction in the number of RT treatments with fewer side effects.

(4) $$SER=\frac{Survival\;fraction\;without\;AuNPs\;(Control))}{Survival\;fraction\;treated\;with\;AuNPs\;(AuNP\;Treated)}$$

#### 4.4.5. Apoptosis and Necrosis

Annexin V–CF Blue/7-aminoactinomycin D (7-AAD) allows the discrimination of early apoptotic cell populations from late apoptotic or necrotic cells through flow cytometry analysis. Annexin V/7-AAD (ab214663, Abcam) was used according to the manufacturer’s protocol to detect and quantify death cell after cells received different treatments for 24 h. Viable cells were double negative, early apoptotic cells were positive to Annexin V and negative to 7-ADD, while late apoptotic cells were double positive and necrotic cells were negative to Annexin V and positive to 7-ADD. Cells were treated with three fractions of 2.5 Gy. Subsequently, cells were detached, washed twice with PBS, resuspended in 1x annexin-binding buffer, and incubated with 5 μL Annexin V-FITC and 5 μL 7-AAD for 15 min at 37 °C. Events were acquired with a FC500 cytometer (Beckman Coulter Life Sciences, Indianapolis, IN, USA), and the acquired data were exported and analyzed with Flowjo software v10.4.2 (FlowJo LLC, Ashland, OR, USA). A total of 50,000 cells were evaluated. 

#### 4.4.6. Reactive Oxygen Species

The reactive oxygen species (ROS) was evaluated by the conversion of 2′,7′–dichlorofluorescin diacetate (DCFDA/H2DCFDA) to 2′,7′–dichlorofluorescein (DCF), a fluorescent compound (Kit ab113851, Abcam) by the presence of ROS. Briefly, 2.5 × 10^4^ cells/well were cultured then washed with buffer and stained with DCFDA for 45 min at 37 °C in the dark. Next, cells were rewashed in buffer and treated with 0.1 mM of AuNPs-PEG. After 24 h, cells were immediately analyzed on a fluorescence plate reader at excitation/emission of 485/535 nm in endpoint mode in the presence of buffer.

### 4.5. Cell Irradiation/Irradiation Setup

**Figure 9:**
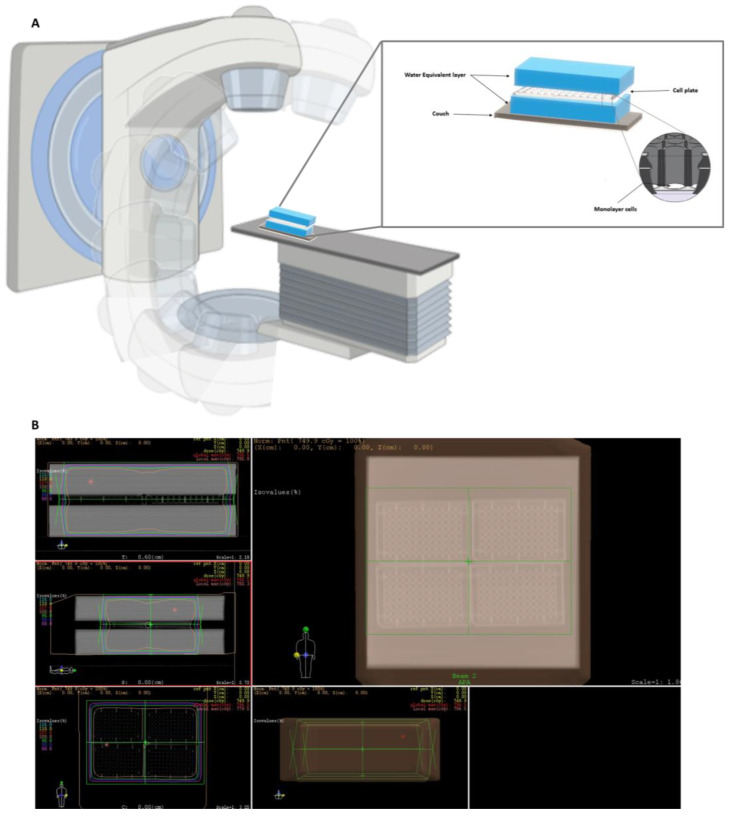
(**A**) Schematic diagram of cell irradiation setup with 6 MV photon beam; (**B**) representative dosimetric plan using the software XIO-Release version 4.70.02. The dose was prescribed to the isocenter using two treatment fields, and the treatment field was covered with different isodose lines, including 100% of the prescribed amount. Pink: 80% isodose line; Dark blue: 90% isodose line; Green: 95% isodose line; Orange: 100% isodose line; Red: 101% isodose line; Yellow: 110% isodose line; Cyan: 115% isodose line.

To calculate the density of the 6-, 24- and 96-well plates, computed tomography scans were performed to obtain three-dimensional (3D) images. A water phantom with a thickness of 5 cm was placed on top and under the plates to simulate a biological structure and provide sufficient backscatter radiation to form an electric equilibrium (Figure 9A).

The dosimetric plan was performed using the software XIO-Release version 4.70.02 and was prescribed to the isocenter, using two fields (one anteroposterior and one posteroanterior, Figure 9B). The fractionation applied was 2.5 Gy in three days until a total radiation dose of 7.5 Gy was completed. Cells were irradiated with a 6 MV photon beam generated by PRIMUS linear accelerator (Siemens) in the radiotherapy department in Centro Hospitalar de São João (Porto, Portugal). The control group did not receive IR. 

### 4.6. Statistical Analysis

All experiments were performed in triplicate. The results are presented as mean  ±  standard deviation (SD). Data were analyzed with Prism 8.0 (GraphPad Software, CA). Differences between treatments were evaluated by Student’s *t*-test or two-way ANOVA with Sidak multiple comparisons test, in accordance with the number of conditions and treatments. Results were considered significant when * *p* < 0.05, ** *p* < 0.01, *** *p* < 0.001 and **** *p* < 0.0001.

## 5. Conclusions 

In conclusion, we demonstrated AuNPs to be potential IR enhancing agents and to show distinct responses depending on the cell lines. AuNP_st_-PEG and AuNP_r_-PEG decreased cellular viability in a dose-dependent manner. Our results showed a tendency of AuNP_st_-PEG and AuNP_r_-PEG to reduce migration and colony formation with and without IR. However, more studies in vitro are required to better understand the cellular mechanism responses and the AuNPs mechanism for radiosensitization. Additionally, AuNP studies with human blood samples should be performed to evaluate the thrombogenicity and hemocompatibility of AuNPs. Taking into account the results obtained in our study, in vivo studies should be performed in the future to evaluate the effects of AuNP treatments on prostate cancer animal models. Studies have reported that AuNPs can produce a wide variety of adverse reactions, impacting numerous organs, including skin, mucosa, kidney, blood, bone marrow, lungs, the nervous system, and the liver. Ultimately, if all trials are correctly evaluated and demonstrate promising results, human clinical research trials should also be considered from phases 1 to 3. In the future, AuNPs can be used in clinical practice, lessening side effects, while safety concerns should always be considered before clinical implementation.

## Figures and Tables

**Table 1:** Hydrodynamic diameters and zeta potential of AuNP_sp_-PEG, AuNP_st_-PEG and AuNP_r_-PEG.

Sample	Hydrodynamic Diameter (nm)	Polydispersity Index (PDI)	Zeta Potential (mV)
AuNP_sp_-PEG	146.73 ± 4.24	0.24 ± 0.005	−5.7 ± 7.6
AuNP_st_-PEG	109.61 ± 1.27	0.14 ± 0.01	33.1 ± 12.0
AuNP_r_-PEG	8.47 ± 0.22 54.58 ± 0.34	0.45 ± 0.01	11.0 ± 18.9

**Table 2:** Comparison of the sensitization enhancement ratio (SER) measured for AuNP_sp_-PEG, AuNP_st_-PEG, AuNP_r_-PEG under irradiation of 2.5, 5, and 7.5 Gy of 6 MV photon beams for PC3, DU145 and LNCaP cell lines.

	Cumulative Dose (Gy) (3 Fractions of 2.5 Gy)	AuNP_sp_-PEG	AuNP_st_-PEG	AuNP_r_-PEG
PC3 Cell Line				
	0	1.43	1.60	0.95
	2.5 (1 × 2.5)	0.88	2.09	1.28
	5 (2 × 2.5)	1.33	1.65	1.75
	7.5 (3 × 2.5)	2.5	1.67	1.67
DU145 cell line				
	0	1.05	1.0	0.89
	2.5 (1 × 2.5)	1.23	1.09	0.66
	5 (2 × 2.5)	0.84	0.91	0.69
	7.5 (3 × 2.5)	1.12	1.64	1.19
LNCaP cell line				
	0	1.4	1.4	0.8
	2.5 (1 × 2.5)	1.2	3	-
	5 (2 × 2.5)	-	1	0.5
	7.5 (3 × 2.5)	-	-	-

## Data Availability

All data that support the findings of this study are available within the article or from the corresponding authors upon reasonable request.
